# Semi-continuous dielectrophoretic separation at high throughput using printed circuit boards

**DOI:** 10.1038/s41598-023-47571-1

**Published:** 2023-11-24

**Authors:** Jasper Giesler, Laura Weirauch, Georg R. Pesch, Michael Baune, Jorg Thöming

**Affiliations:** 1 Chemical Process Engineering, Faculty of Production Engineering, University of Bremen, Bremen, Germany; 2 School of Chemical and Bioprocess Engineering, University College Dublin, Dublin, Ireland; 3 Center for Environmental Research and Sustainable Technology (UFT), University of Bremen, Bremen, Germany

**Keywords:** Engineering, Materials science

## Abstract

Particle separation is an essential part of many processes. One mechanism to separate particles according to size, shape, or material properties is dielectrophoresis (DEP). DEP arises when a polarizable particle is immersed in an inhomogeneous electric field. DEP can attract microparticles toward the local field maxima or repulse them from these locations. In biotechnology and microfluidic devices, this is a well-described and established method to separate (bio-)particles. Increasing the throughput of DEP separators while maintaining their selectivity is a field of current research. In this study, we investigate two approaches to increase the overall throughput of an electrode-based DEP separator that uses selective trapping of particles. We studied how particle concentration affects the separation process by using two differently-sized graphite particles. We showed that concentrations up to 800 mg/L can be processed without decreasing the collection rate depending on the particle size. As a second approach to increase the throughput, parallelization in combination with two four-way valves, relays, and stepper motors was presented and successfully tested to continuously separate conducting from non-conducting particles. By demonstrating possible concentrations and enabling a semi-continuous process, this study brings the low-cost DEP setup based on printed circuit boards one step closer to real-world applications. The principle for semi-continuous processing is also applicable for other DEP devices that use trapping DEP.

## Introduction

Particle separation is a prominent unit operation in engineering and is used in processes ranging from nanotechnology^1,2^ to biotechnology^3^, recycling^4,5^ and the processing of minerals^6–8^. To address the plurality of separation problems, a variety of approaches, such as flotation^7,9^, centrifugation^4,10^, field-flow fractionation^11^ or filtration^12^ were developed.

One force that can be used to manipulate particles in the micrometer or sub-micrometer size range is dielectrophoresis (DEP). The dielectrophoretic movement occurs when a polarizable particle is subjected to an inhomogeneous electric field. This field can be generated with direct or alternating current. The bandwidth of devices that utilize DEP ranges from industrial scale separators with throughput of up to hundreds of cubic meters per min but low selectivity^13,14^ to microfluidic separators that feature flow rates of only a few microliter per hour but can resolve minute differences in the size of DNA^15^. Potential applications of DEP, aside from biotechnology where it is well studied^3,16–18^, could be the sorting of carbon nanotubes according to their conductivity^19^ or the recycling of lithium-ion batteries^20^. To achieve a combination of high selectivity and high throughput is subject of current research. In the recent past, our group^20–28^ among others^29–31^, aimed at achieving this desirable combination.

DEP separators can, for example, be classified with respect to their electrode configuration. Here, it can be distinguished between insulator-based (iDEP) and electrode-based (eDEP) setups. In an iDEP separator, the electric field is scattered by adding an insulating material into the field which gives rise to high field gradients that are required for DEP. In contrast, eDEP devices feature electrodes that create an inhomogeneous electric field due to their placement or geometry. Another type of classification of DEP setup is whether the process is continuous or discontinuous^32^. In continuous processes, for example, different particles types are constantly directed from one inlet into specific outlets which is called streaming DEP^33,34^ or continuous separation. In a discontinuous process, particles are (selectively) trapped and remobilized afterward. This is called trapping DEP. On the one hand, the concept of trapping DEP was successfully upscaled by using iDEP^21,23,24,28^ and recently also eDEP approaches^27^ while maintaining selectivity. On the other hand, the trapping limits throughput as during the remobilization no particles can be separated. In this operational mode, throughputs of up to around 11 mL/min could be achieved with approaches that allow further scaling. In comparison, in 2022, Faraghat et. al.^30^ presented and validated a concept that features streaming DEP at a volume flow 0.5 mL/min.

In this study, we investigate how the throughput of trapping eDEP devices can be further increased without increasing the volume flow. This can be done by increasing concentration of the particles in the feed suspension or by reducing the time that is required to remobilize previously trapped particles. The variation of concentration is conducted with two differently sized graphite particles. First, Timrex KS6 (MSE Supplies LLC, USA) with $d_{50}=3.4$ $\upmu \hbox {m}$ and second, the bigger C-NERGY Actilion GHDR 15-4 (Imerys Graphite & Carbon, Switzerland) with $d_{50}=17$ $\upmu \hbox {m}$ are used. The reduction of the remobilization time is then investigated by a parallelization of two identical channels in combination with 4-way valves, stepper motors and a microcontroller. This allows to perform semi-continuous fractionation, thus overcoming one of the main drawbacks of trapping-based DEP devices.

**Figure 1:**
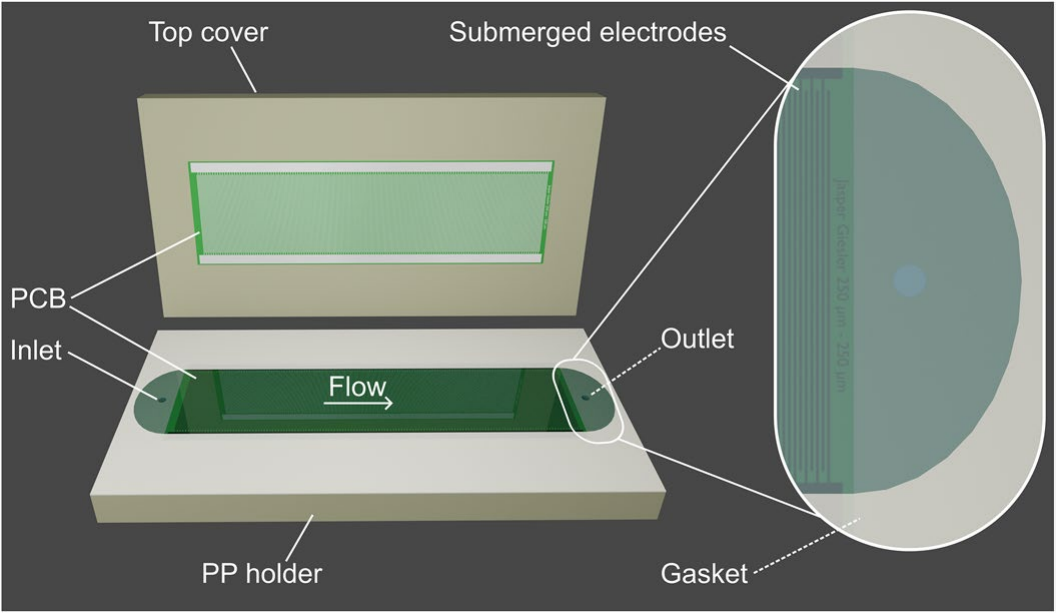
Render of a filtration cell based on printed circuit boards (PCBs) as it is used in this study. Electrode arrays on PCBs form ceiling and bottom of the channel. The electrodes are glued into polypropylene (PP) holders and are electrically connected via wires at the back (not shown). A gasket with a thickness of 0.5 mm acts as spacer and wall of the channel. The electrodes have a width and spacing of 250 $\upmu \hbox {m}$ and the PCBs have a dimension of $45\times 150$ mm. Through the channel a particle suspension is pumped from inlet to outlet.

The discontinuous yet high throughput eDEP approach used in this study is portrayed in Fig. 1. The device was presented in previous studies and is capable of handling volume flows of around 10 mL/min, selective trapping, and also is applicable for conductive particles^20,27^. The device features two low cost custom-designed printed circuit boards (PCBs) that face each other and form ceiling and bottom of the channel similar to a device for static measurements presented by Burt, Al-Ameen and Pethig in 1989^35^. On the PCBs, an interdigitated electrode array with a width and spacing of 250 $\upmu \hbox {m}$ is patterned. A 0.5 mm gasket acts as sealing and spacer between the two PCBs to create a channel. The PCBs have a size of $45\times 150$ mm. A suspension is pumped from an inlet to an outlet and particles are attracted toward the electrodes by positive DEP (pDEP) or repelled from them by negative DEP (nDEP).

Whether a particle experiences pDEP or nDEP, depends on the real part of the so-called Clausius-Mossotti factor $\text{Re}(CM)$. For an homogeneous and spherical particle, $\text{Re}(CM)$ ranges from $-0.5$ to 1.0 and can be calculated as^36^,

1 $$\begin{aligned} \text{Re}(CM)=\text{Re}\left( \frac{\tilde{\varepsilon }_\text{p}-\tilde{\varepsilon }_\text{m}}{\tilde{\varepsilon }_\text{p}+2\tilde{\varepsilon }_\text{m}} \right) . \end{aligned}$$

Here, the index *p* represents properties of the particle and *m* of the medium. The complex permittivity ($\tilde{\varepsilon }=\varepsilon _0\varepsilon _\text{r}-i\frac{\sigma }{\omega }$) incorporates the relative permittivity $\varepsilon _\text{r}$, the permittivity of the vacuum $\varepsilon _0$, and the angular frequency of the electric field $\omega$ in combination with the conductivity of a material $\sigma$. If $\text{Re}(CM)$ is negative particles experience nDEP and are repelled from local field maxima. If $\text{Re}(CM)$ is positive, the particles experience pDEP and are attracted towards local field maxima. The DEP force itself, $\mathrm {{\textbf {F}}}_\text{DEP}$, for a homogeneous and spherical particle can be approximated by^36,37^,

2 $$\begin{aligned} \mathrm {{\textbf {F}}}_{\text{DEP}}=2\pi r^3_\text{p} \varepsilon _\text{m} \text{Re}(CM) \nabla \mathrm {|{{\textbf {E}}_{rms}}|}^2 \end{aligned}$$

with the electric field $\mathrm {{\textbf {E}}_{rms}}$ and $r_\text{p}$ as the radius of the particle.

## Results and discussion

The results are grouped into two subsections. First, the influence of the concentration on the performance of the device for both types of graphite is investigated. Second, the concept for semi-continuous fractionation is presented and tested. We examine the semi-continuous fractionation by using KS6 graphite and similar sized 3 $\upmu \hbox {m}$ polystyrene (PS) fluorescent particles. At the selected frequency of 500 kHz, graphite shows pDEP and PS nDEP allowing for a semi-continuous material-selective separation. These two particles are first tested separately and afterward as a mixture. We suspended all particles in this study in an aqueous medium with a conductivity of 3 $\upmu \hbox {S/cm}$. Piston pumps generate a volume flow that is constantly entering the channel. The channel is connected to a spectrometer to measure the particle concentration at the outlet. For details on the experimental procedure, particles, voltage generation, data acquisition and processing, please consult the "Methods" section. As the graphite particles in this study are distributed in shape and size (Supplementary Figs. 1 & 2), the exact scatter properties of these particles are unknown. Therefore, the optical measurements can not be interpreted quantitatively. However, we aim for complete removal of the graphite particles, which is qualitatively fairly simple to determine using reflection measurements.

### Influence of concentration

**Figure 2:**
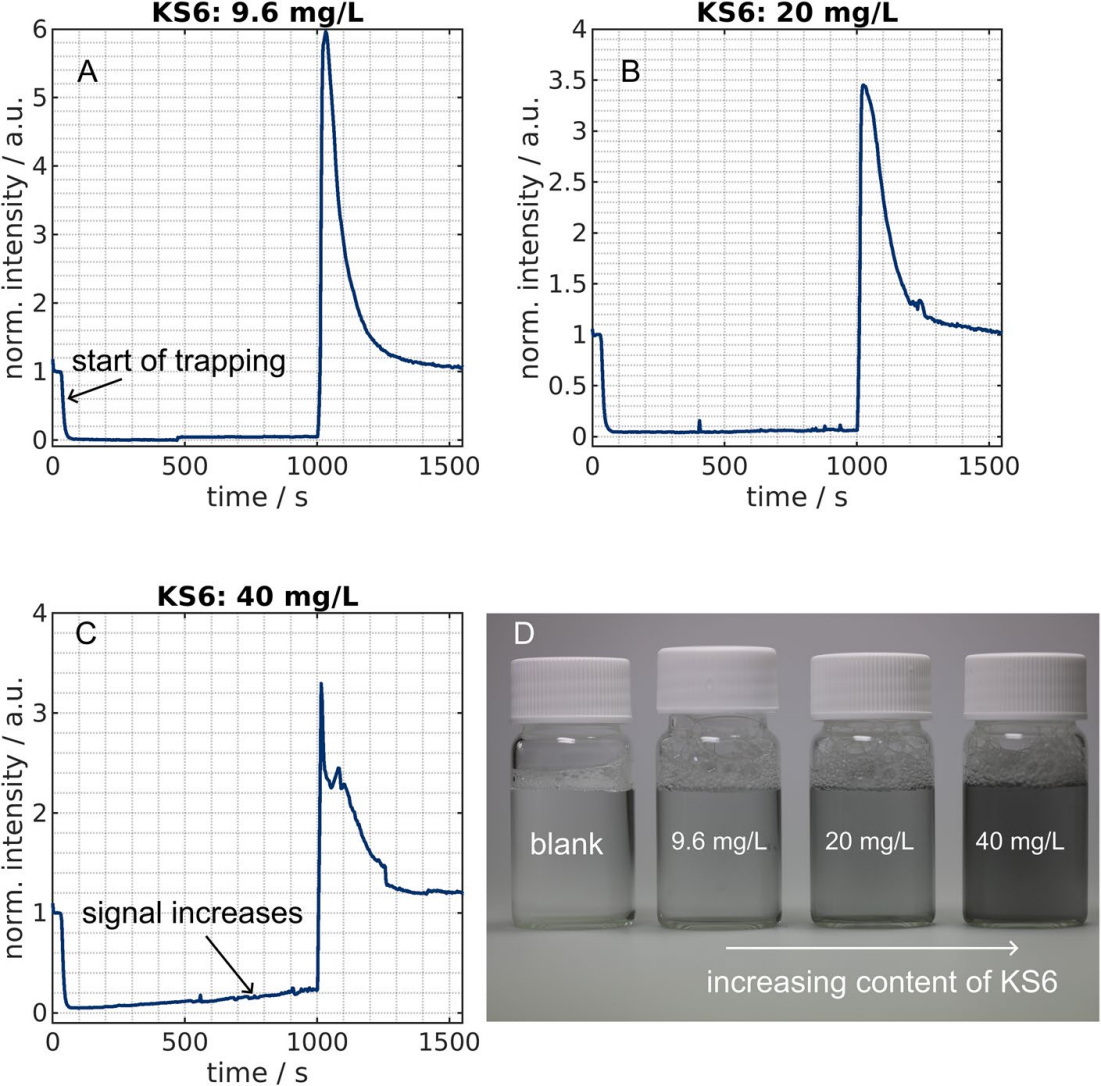
Normalized intensity over time for KS6 graphite with a loading of 9.6 mg/L (**A**), 20 mg/L (**B**) and 40 mg/L (**C**). A sinusoidal voltage with 75 V$_\text{pp}$ and a frequency of 500 kHz was turned on after 30 s and switched off at 1000 s. The illustrated graphs are mean values of three experiments. Additionally, a photograph of flasks (**D**) with 15 mL of the different particle concentrations is shown in comparison to $1\%$ Tween 20 in pure water (blank) which is the solution in which the particles were suspended.

In a previous study^20^, we found that for the smaller graphite particles (KS6) a voltage of 75 V$_\text{pp}$ at 500 kHz and 6 mL/min results in a complete removal of graphite from the suspension. In contrast, 30 V$_\text{pp}$ at 500 kHz were sufficient to remove larger graphite particles (Actilion) completely from the suspension. Consequently, we selected 75 V$_\text{pp}$ and 30 V$_\text{pp}$ for KS6 and Actilion, respectively, as process parameters to investigate the effect of concentration. In both cases, experiments were carried out at a field frequency of 500 kHz and a volume flow of 6 mL/min.

We reproduced the experiment presented by Giesler et al.^20^ by using the same concentration of KS6 as in that study (9.6 mg/L) (Fig. 2A). As previously, a complete removal of the smaller graphite could be observed for as long as the electric field was generated ($t=30$ s to $t=1000$ s). The displayed intensity over time plots show averaged data from three experiments and the intensity is normalized to the initial concentration. When the voltage is turned on after 30 s, a clear drop of the intensity can be observed that reaches the background intensity and stays there until the voltage is turned off again. The intensity then exceeds the initial level of 1 significantly only to drop afterward to the initial concentration again. The observable peak corresponds to the remobilization of previously trapped particles which then exit the channel and create high reflection signals. At about 500 s, a discontinuity of the signal is visible. The plotted data is as mentioned an average of three experiments. The jump corresponds to one of the three experiments and is not visible in the other two. Summarizing, at 9.6 mg/L at the selected trapping duration, which is over 15 minutes, no saturation effect is visible.

We then increased the concentration of KS6 to 20 mg/L (Fig. 2B) and 40 mg/L (Fig. 2C). At 20 mg/L, no apparent changes were visible compared to the first concentration and thus, again, no saturation effect could be observed. In contrast, at the highest tested concentration (40 mg/L), we found an increase in intensity during the trapping after a short period of time. While the signal drops also to background signal at the beginning, it starts to raise in the following. As particles are continuously entering the channel and the intensity is still below unity, this does correspond to a reduced trapping and not to a release of particles. We conclude that at this concentration a saturation effect becomes visible. The separation efficiency decreases with time, thus, a higher concentration than 40 mg/L seems unfeasible for this particle type. In the past, we tested higher concentrations with other particles (Actilion). Consequently, this can not be the absolute limit with respect to mass of the separator. To further evaluate this point, we conducted experiments with the significantly larger Actilion graphite particles at higher concentrations.

**Figure 3:**
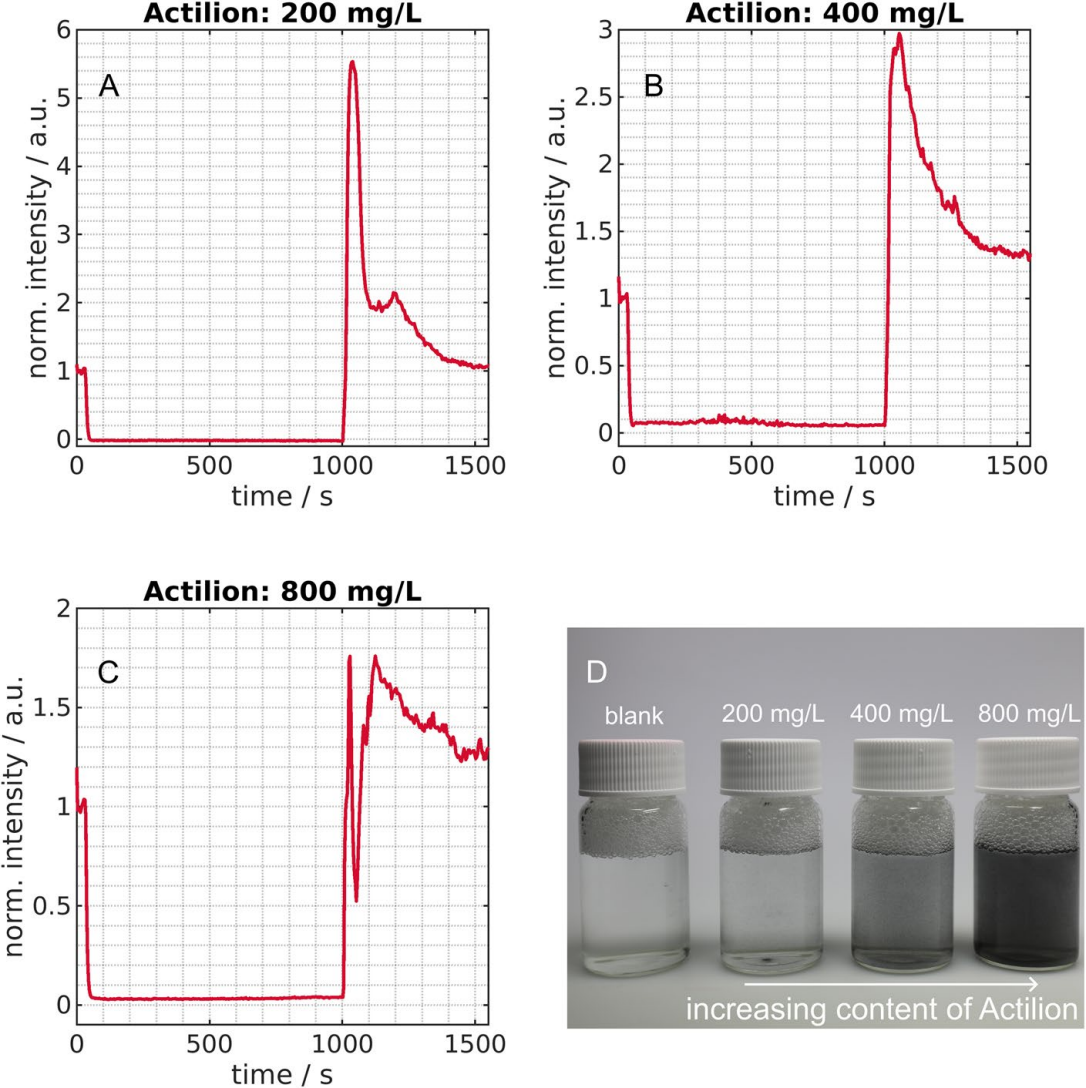
Normalized intensity over time for Actilion graphite with a loading of 200 mg/L (**A**), 400 mg/L (**B**) and 800 mg/L (**C**). A sinusoidal voltage with 30 V$_\text{pp}$ and a frequency of 500 kHz was turned on after 30 s and switched off at 1000 s. The illustrated graphs are mean values of three experiments. Additionally, a photograph of flasks (**D**) with 15 mL of the different particle concentrations is shown in comparison to $1\%$ Tween 20 in pure water (blank) which is the solution in which the particles were suspended.

The results from these experiments are displayed in Fig. 3A–C. When comparing the photographs of the mixtures (Figs. 2D and 3D), the turbidity per mass of the KS6 is much more pronounced due to KS6’s higher specific surface area. At Actilion concentrations of 200 mg/L, 400 mg/L and 800 mg/L the setup showed nearly complete removal of the Actilion graphite. At 200 mg/L (Fig. 3A) the background intensity is reached during trapping (normalized intensity$=0$), indicating complete Actilion removal. At higher concentrations (Fig. 3B and C), the intensity does not drop to entirely to 0. Nonetheless, in contrast to the smaller particles, no distinct saturation effect is observable in these experiments. This is likely to the higher average mass per particle of Actilion compared to KS6 and the stronger DEP force on the larger particles (Eq. 2). However, we found that handling of Actilion is challenging at 800 mg/L as the particles accumulate in the flow cuvette. We also observed that such high concentrations can damage the electrodes, likely due to short circuits. These damages occurred when cleaning of the electrodes with a high fluid flow rate in between of the experiments is incomplete. Due to these difficulties, we decided not to test higher concentrations. In the intensity over time plot for the 800 mg/L (Fig. 3D), during the remobilization ($t>1000$ s), a sharp decrease is visible despite the fact that particles were eluted from the channel. The reason is unclear but it may be linked to too many particles present in the flow cuvette and consequently low total reflection signal. We additionally observed in the experiments that the maximum level of the spectrometer was reached during the remobilization at some wavelength (e.g. Supplementary Fig. 4) at these high concentrations directly before this phenomenon. This again indicates the presence of many particles in the cuvette.

Concluding, for this separator, a maximum of 40 mg/L for the KS6 graphite particles and of 800 mg/L for the larger Acitlion particles was found. This difference is likely linked to different strong DEP responses of the individual particles and on the area one particle covers per mass.

### Semi-continuous separation

Increasing the concentration is not the only way to increase throughput. In the following subsection, a semi-continuous separation approach is presented. In contrast to a completely continuous separation, here, two devices running in parallel are mimicking a continuous operational mode. To distinguish the approach presented here from streaming DEP, the term semi-continuous process is used. First, the apparatus for semi-continuous fractionation is introduced, afterward the performance of the device is tested with particles that show pDEP (KS6 graphite) and nDEP (3 $\upmu \hbox {m}$ PS, Polysciences Europe GmbH, Germany). The performance of the device is first monitored for both particles separately and as a mixture subsequently. For the graphite a concentration of 9.6 mg/L was used and around 7.9 mg/L for the PS which is equal to 150 $\upmu \hbox {L}$ of a $2.5\%$ stock suspension suspended in 500 mL medium.

**Figure 4:**
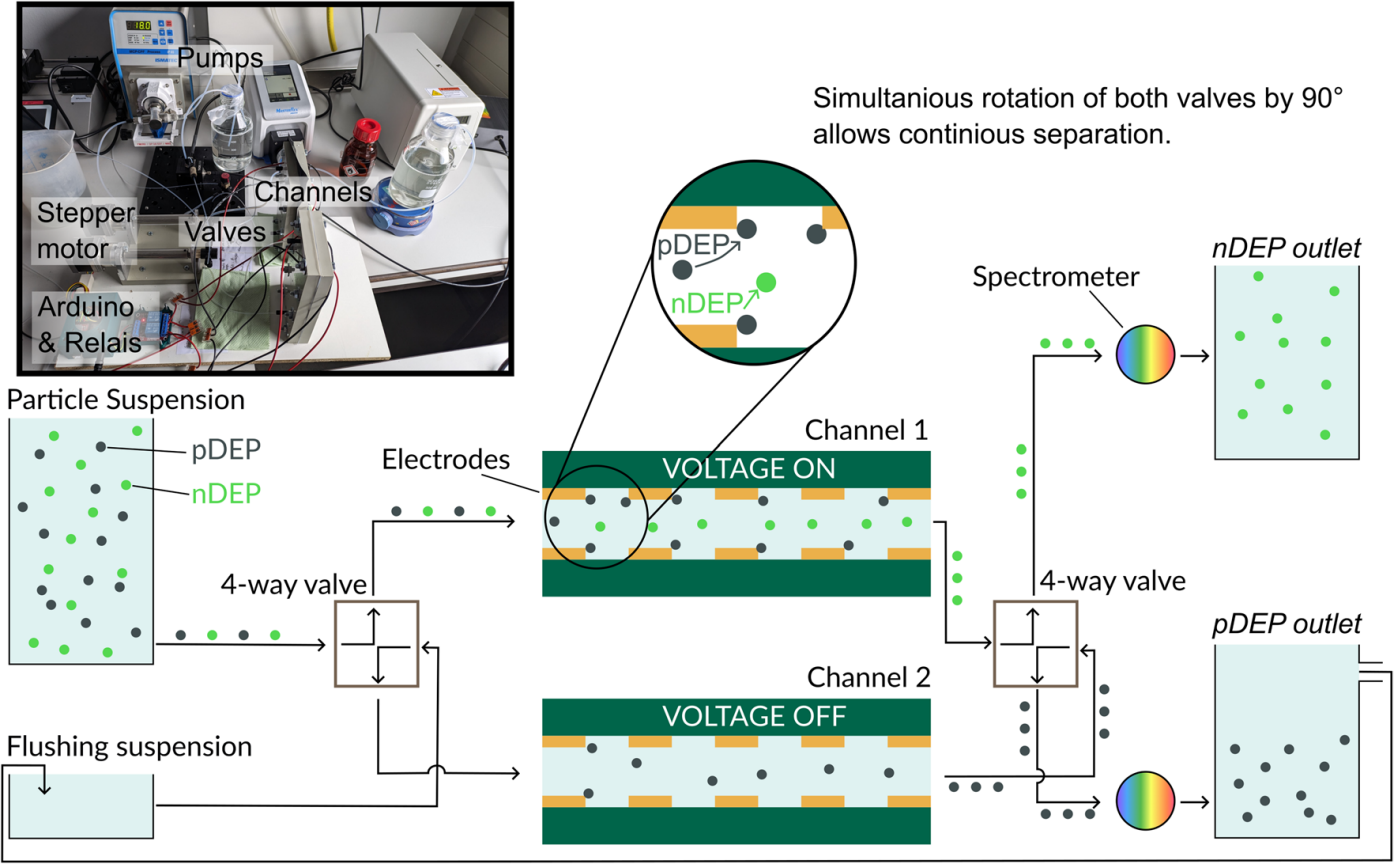
Sketch and photograph (insert) of the setup for semi-continuous separation. Two channels are connected to two 4-way valves. The left valve controls into which of the two channels the particle suspension is pumped. Further, by adjusting the right valve, the fluid flow can be directed into one of the two outlets. The valves are actuated by stepper motors. By this configuration, the position of the valves can be adjusted by an Arduino Uno microcontroller. The Arduino also controls two relays and thus, when rotating the valves, can also switch on and off the voltage for each channel. Right before particles reach one of the two outlets, a reflection or fluorescence spectrum can be recorded using a spectrometer. When a sufficiently high voltage is applied, only particles that show no DEP or nDEP will reach the upper outlet (*nDEP outlet*). Whereas at the lower outlet (*pDEP outlet*), previously trapped particles can be found. To reduce the amount of waste produced in the experiments, one can flush with the suspension obtained from *pDEP outlet*.

#### Concept

The procedure for the semi-continuous separation is illustrated in Fig. 4. In order to achieve a continuous fractionation, two channels were combined with two 4-way valves. These valves were attached to two stepper motors (NEMA17-05GM, reichelt elektronik GmbH & Co. KG, Germany), a microcontroller (Arduino Uno) and an Arduino motor shield (ARD-CNC-KIT1, reichelt elektronik GmbH & Co. KG, Germany). One valve was connected to the pumps and the inlets of the two channels whereas the other valve was connected to the outlet of the channels, and the reservoirs termed *pDEP* and *nDEP outlet*.

During the experiments, one channel is fed with particle suspension and at the same time a voltage is applied to this channel. Thus, particles that show pDEP will get immobilized in contrast to less polarizabile particles that show nDEP or no DEP. These particles are eluted into the outlet termed *nDEP*. At the same time, a second channel is connected to a flushing suspension and is not connected to a power source. Thus, previously trapped particles can be remobilized and flushed into the *pDEP outlet*. By rotating the valves by $90^\circ$ using the stepper motors and switching the voltage signal from the first channel to the second via relays, the pumps and outlets are now connected to the other channel.

Experiments were conducted with a sinusoidal voltage with 75 V$_\text{pp}$ at a frequency of 500 kHz. The voltage was constantly turned on after 30 s which allowed to record the initial particle concentration in the time frame before. Valves and relays were actuated every 300 s and firstly at $t=330$ s. The volume flow of the particle suspension was set to 6 mL/min while the flow of the flushing suspension was 3 times higher to achieve good remobilization and resulted to be 18 mL/min. In order to reduce the amount of produced wastewater, the flask containing the flushing suspension is also the *pDEP outlet*. Thus, particles, mostly those that show pDEP, will accumulate within this suspension. As only pDEP particles are supposed to reach this outlet, this is considered unproblematic. By drawing the suspension at the top of the flask while not stirring the vessel, sedimentation counteracts the accumulation to some extent.

#### Monodisperse experiments

**Figure 5:**
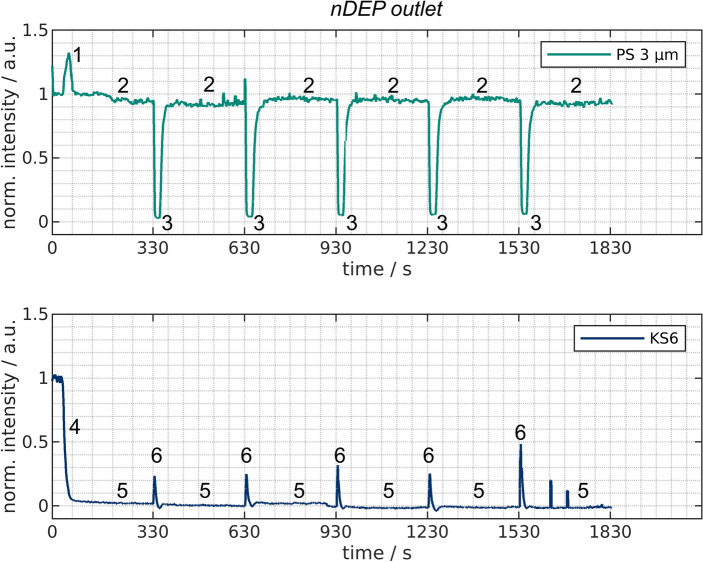
Normalized intensity over time at the *nDEP outlet* for experiments with only PS (top) or KS6 graphite (bottom) present in the channels. This outlet is connected to the channel where a voltage is applied and particle suspension is pumped through. The numbers correspond to (reoccurring) events. These events are the remobilization of previously trapped particles (1), negligible trapping of PS (2), actuation of the valves (3 &6), start of the trapping of KS6 when applying a voltage (4), and steady-state trapping of KS6 (5).

With only one particle type present per experiment, the evaluation of these experiments is straight forward. Only one outlet at a time is monitored with a spectrometer. The other outlet is monitored in an additional run. Figure 5 shows the intensity over time recorded at the *nDEP outlet* for KS6 and PS. This outlet belongs to the currently powered channel and thus, particles that show strong pDEP are retained and other particles are eluted. We expect KS6 to show pDEP and PS to show nDEP at the selected process parameters (medium conductivity and frequency). When looking at the plot for PS, one can see a peak as soon as the voltage is turned on (1) for the first time. This is likely linked to PS particles that show nDEP and where previously somewhere attached or sedimented inside the channel. Afterward, a low yet observable signal reduction can be observed (2) which is below $10\%$. The clear majority of the PS particles, consequently, leaves the channel despite the presence of the electric field, which indicates that these particles show nDEP behavior. As soon as the valves are actuated (3), the other channel is now flushed with particle suspension which, however, previously was cleaned with the flushing suspension. This flushing suspension has few or none particles in it and thus a reflection/fluorescence signal close to the background intensity. The signal than rises rapidly when the particles reach the outlet again.

In comparison to the PS particles, the graphite particles show substantial pDEP. This becomes obvious as soon as the voltage is turned on. The intensity drops quickly (4) and reaches a level close to the background signal (5) at which the signal remains. This indicates high removal of the graphite particles which is the exact opposite behavior of the PS particles. When the valve is actuated (6) a small peak becomes visible in the plot. This peak is the result of those particles that can not be trapped in the period of switching voltage and valves. It is because the switching of the valves takes about 1 s. This is due the fact that the motors need to perform 259 steps to achieve a rotation by $90^\circ$. We found that a pause of 2 ms per step was necessary in order to achieve high reproducibility as otherwise sometimes steps are left out which results in incomplete rotation. We think that an optimized switching protocol might avoid the peaks observed at (3) and (6) in Fig. 5. Summarizing, the *nDEP outlet* showed that the vast majority of PS is reaching this outlet in contrast to the graphite particles.

**Figure 6:**
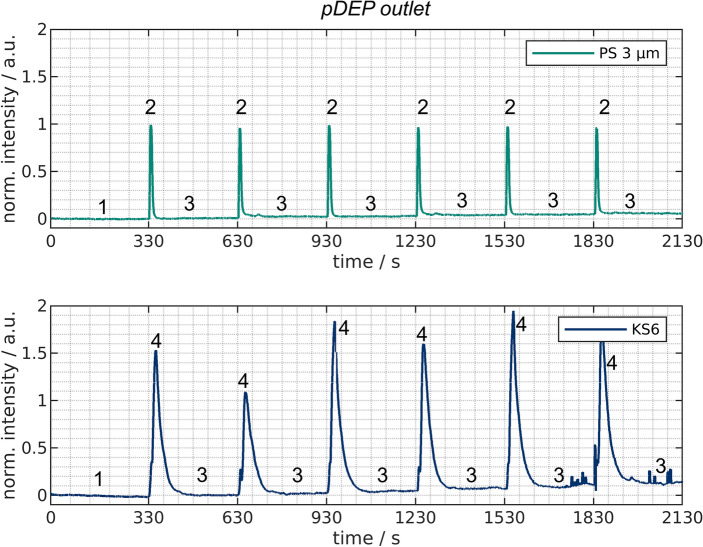
Normalized intensity over time at the *pDEP outlet* for experiments with only PS (top) or KS6 graphite (bottom) present in the channels. This outlet is connected to the channel where no voltage is applied and flushing suspension is circulated. The numbers correspond to (reoccurring) events. These events are the circulation of pure flushing suspension (1), actuation of the valve (2 & 4) and the new steady-state signal of the flushing suspension which can increase over time due to the accumulation of particles.

To complete the picture, the results of the *pDEP outlet* are shown in Fig. 6. Here, the outlet of the channel without applied voltage is connected to the spectrometer and the channel is flushed with the flushing suspension at 18 mL/min. For the first 330 s only the background intensity is observable (1) as at the beginning no particles can be remobilized. When the valves are actuated (2), PS particles reach this outlet at almost the initial concentration within the feed suspension. This is because of the switching times already mentioned above. The peak observed at (2) in Fig. 6 thus is due to the particles that are present in the channel when the valves are actuated. The peak is thin and after a few seconds the new steady-state value is reached (3). When looking at the value over time it rises slightly, as the suspension leaving the *pDEP outlet* is reused as the flushing suspension.

For the graphite particles, again, during the first cycle (1), only background is visible. As soon as the valves rotate, previously trapped particles are released (4). In comparison to the PS particles, the peak exceeds unity and is substantially broader. This is due to the previously trapped particles and their subsequent detachment when turning off the voltage. The steady-state level (3) reached after release rises faster and stronger compared to the PS particles due to a greater accumulation of the graphite particles in the *pDEP outlet*.

These experiments showed that a semi-continuous processing is possible. Particles that show pDEP or nDEP are continuously sorted into different outlets. In contrast to streaming DEP, however, two separation columns are required. Thus, the process differs from continuous separation. It should be noted that the undesirable peaks (2) in Fig. 6 and (3) and (6) in Fig. 5 and their influence is decreasing with increasing cycle time as then more particles reach the desired outlet. By optimizing the cycle length but also the switching protocol, e.g. by switching the valves connected to the outputs later than the other one, further improvements should be achievable without much effort. This, however, is beyond the scope of this study.

#### Mixture

**Figure 7:**
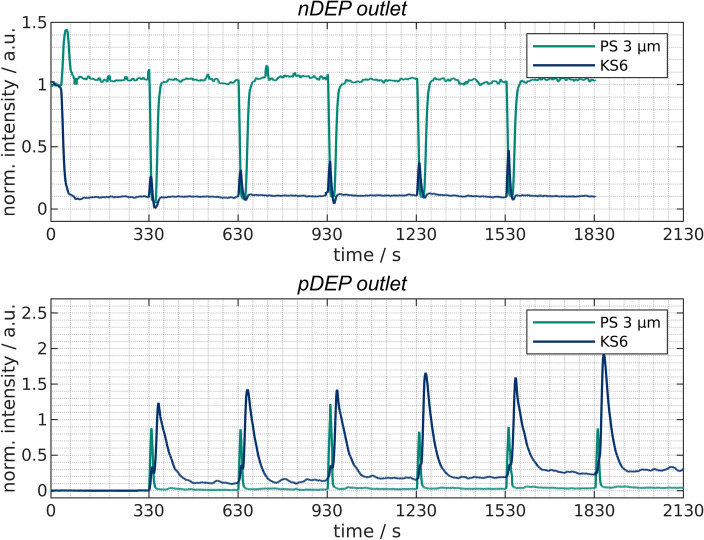
Normalized intensity over time at the *nDEP outlet* (top) and the *pDEP outlet* (bottom) for experiments with mixtures of PS and KS6 graphite present in the channels.

As final step, we conducted an experiment with a mixture of graphite and polystyrene using the same concentrations as before. Now both particle types are suspended, increasing the overall loading. The result of this experiment is one combined fluorescence/reflection spectrum from the PS and the graphite particles. The PS particles, however, show a significant different reflection/fluorescence than the KS6 (Supplementary Fig. 5). When assuming that the overall spectrum is linearly composed out of the single spectra from graphite and PS, a linear unmixing is possible by using a global optimization as we presented in Reference^21^. An example for this is shown in the Supplementary Fig. 5. Supplementary Fig. 6 shows a comparison between the unmixed and mixed spectrum.

At the first glance, both, the *pDEP* and the *nDEP outlet* (Fig. 7) are quite similar to the monodisperse experiments (Figs. 5 and 6). At the *nDEP outlet*, the PS particles are detected, whereas the concentration of KS6 drastically decreases once the voltage is turned on at $t=30$ s. Yet, two differences can be noticed when comparing the *nDEP outlet* plots from monodisperse experiments with those of the mixture. First, the KS6 intensity drops to about $10\%$ of its original value (instead of 0 as in the monodisperse experiments), which indicates that the trapping of KS6 is decreased when PS is present. This might be due to particle-particle interactions. As PS particles are repelled from field maxima but scatter the electric field and produce local field inhomogenities, these might attract graphite particles which are then leaving the channel along with the PS particles. Second, the PS concentration again remains high at the *nDEP outlet*, but seems to stay just above 1. This could be due to the linear unmixing procedure which, as the name suggests, relies on a linear composition of the spectrum. Possibly, some fluorescence from the PS particles is absorbed by the graphite particles which wrongly suggests a lower PS concentration for the first 30 s of the experiments than actually present. However, the deviation is small ($5\%$) and as this study aims to provide a proof-of-principle rather than a quantitative analysis, we decided to include this data.

The *pDEP outlet* again shows the expected behavior and in direct comparison, it is obvious that significantly more KS6 is reaching the *pDEP outlet* than PS particles do. Again, the accumulation of both particles can be seen as the suspension leaving the *pDEP outlet* is used as flushing suspension.

## Conclusion

Overall, this study shows the transferability of the results of tests with monodisperse particle systems to the separation behavior of their mixtures. It further demonstrates that a continuous material-selective fractionation is possible with two channels in parallel by using trapping dielectrophoresis. The procedure presented in this study overcomes the limitations of trapping dielectrophoresis while maintaining its high flow rates.

## Methods

### Device fabrication

The fabrication of the device is described in detail in the references^27^ and^20^. The electrodes have an outer dimension of $45\times 150$ mm and were custom designed by the authors. The PCBs are inexpensive ($<1$ €/pc.) (manufactured by JiaLiChuang (HongKong) Co., Limited, China) and can be reordered by using the manufacturing data available in reference^38^. A 0.5 mm silicon gasket is used as sealing.

The electrodes were glued into two polypropylene holders (PP) and connected via wires to an amplifier (F30PV, Pendulum Instruments, Sweden) which can provide $75~\mathrm {V_{pp}}$ at up to 2 A. A sinusoidal signal is generated using a signal generator (Rigol DG4062, Rigol Technologies EU GmbH, Germany), which is monitored using a power analyzer (PPA1510, Newtons4th Ltd, United Kingdom) and an oscilloscope (Rigol DS2072A, Rigol Technologies EU GmbH, Germany).

### Experimental setup

The measurement setup is described in detail in a previous publication^27^. Briefly, one outlet of the channels is connected to the inlet of a flow cuvette (176-765-14485-40, Hellma GmbH & Co. KG, Germany). The cuvette is placed in a cuvette holder (CVH100/M, Thorlabs GmbH, Germany) into which a liquid light guide (LLG) emits light created by a white-light source (XCite 120 PC, Excelitas Technologies Corp., USA). In $90^\circ$ with respect to the LLG, a light guide behind a triple-bandpass filter (DAPI/FITC/TRITC) is placed which leads to a spectrometer (Silver nova, StellarNet, Inc., USA) and measures light intensity from $190-1100$ nm. A LabVIEW program is used to record the data.

To pump the fluid through the channels, two piston pumps were used (Ismatec MCP-CPF IP65, Cole-Parmer GmbH, Germany and MFLX78018-60, VWR International GmbH, Germany). The solution contained mainly pure water (Omniatap 6 UV/UF, stakpure GmbH, Germany) to which $0.01\%$ Tween 20 (Sigma-Aldrich, Germany) as detergent and KCl were added until the desired conductivity of 3 $\upmu \hbox {S/cm}$ was achieved. The solution was then degassed while stirred using a magnetic stirrer at 70 mbar for several minutes to remove air from the suspension. As particles were added to the feed suspension, it was also constantly stirred to avoid sedimentation.

### Data generation and processing

The experiments for the loading study were repeated three times to ensure statistical validity. For each experiment a new file was generated which is available in the online repository. The experiments for the semi-continuous separation were conducted in such a way that three full cycles are recorded. One cycle includes one trapping and one remobilization step for each channel. As at the *pDEP outlet* no particles are present at the beginning, one additional switching event was recorded. This is not necessary for the *nDEP outlet* as particles are always present here. The three full cycles were recorded in one run and consequently stored in one file. No additional repetitions were made as the experiment already contains the repetitions in itself.

The data was evaluated with in-house developed MATLAB scripts that are also available at in the mentioned repository. The displayed data is smoothed to reduce the noise by a moving average. Prior to the experiments with particles present, a blank suspension is pumped through the channel and recorded as background intensity for about 60 s. This signal was then averaged and subtracted from the data recorded with particles in it. The intensity was normalized on the average intensity that is present before voltage is turned on. Consequently, the plots (except those of the *pDEP outlet*) start around one.

### Supplementary Information

Supplementary Information.

## Data Availability

All measurement data that is included in this publication is uploaded to an online repository^39^ along with MATLAB scripts to evaluate the data, and generate the plots of this study.
