# Supplementary material for: Semi-continuous dielectrophoretic separation at high throughput using printed circuit boards
Source: Sci Rep. 2023 Nov 24;13:20696. doi: 10.1038/s41598-023-47571-1 (PMC10673871; doi:10.1038/s41598-023-47571-1)
Supplement: Supplementary file 1 — Supplementary Information. [file 41598_2023_47571_MOESM1_ESM.pdf]

# Supporting information
# Semi-continuous dielectrophoretic separation at high throughput using printed circuit boards

**Jasper Giesler[1], Laura Weirauch[1], Georg R. Pesch[2], Michael Baune[1,3], and Jorg Thöming[1,3,*]**

[1]Chemical Process Engineering, Faculty of Production Engineering, University of Bremen, Bremen, Germany
[2]University College Dublin, School of Chemical and Bioprocess Engineering, Dublin, Ireland
[3]Center for Environmental Research and Sustainable Technology (UFT), University of Bremen, Bremen, Germany
[*]thoeming@uni-bremen.de

## ABSTRACT

Particle separation is an essential part of many processes. One mechanism to separate particles according to size, shape, or material properties is dielectrophoresis (DEP). DEP arises when a polarizable particle is immersed in an inhomogeneous electric field. DEP can attract microparticles towards the local field maxima or repulse them from these locations. In biotechnology and microfluidic devices, this is a well-described and established method to separate (bio-)particles. Increasing the throughput of DEP separators while maintaining their selectivity is a field of current research. In this study, we investigate two approaches to increase the overall throughput of an electrode-based DEP separator that uses selective trapping of particles. We studied how particle concentration affects the separation process by using two differently-sized graphite particles. We showed that concentrations up to 800 mg/L can be processed without decreasing the collection rate depending on the particle size. As a second approach to increasing the throughput, parallelization in combination with two four-way valves, relays, and stepper motors was presented and successfully tested to continuously separate conducting from non-conducting particles. By demonstrating possible concentrations and enabling a semi-continuous process, this study brings the low-cost DEP setup based on printed circuit boards one step closer to real-world applications. The principle for semi-continuous processing is also applicable for other DEP devices that use trapping DEP.

## 1 Further information on the particles

The graphite particles used in this study are have a distribution in shape and size. The Actilion graphite are substantially larger than the KS6 particles and are *potato* shaped as can be seen in Figure 1. In contrast, the KS6 particles appear more as flakes as can be seen in Figure 2. The PS particles, have a very narrow size distribution and, in contrast to graphite, show nDEP at 500 kHz as it is displayed in Figure 3.

High concentrations of the particles inside of the flow cuvette can lead to such a high reflection signal that the sensor of the spectrometer can not resolve it properly. In this case, the maximum intensity of the sensor is stored in the data files which can be seen in Figure 4.

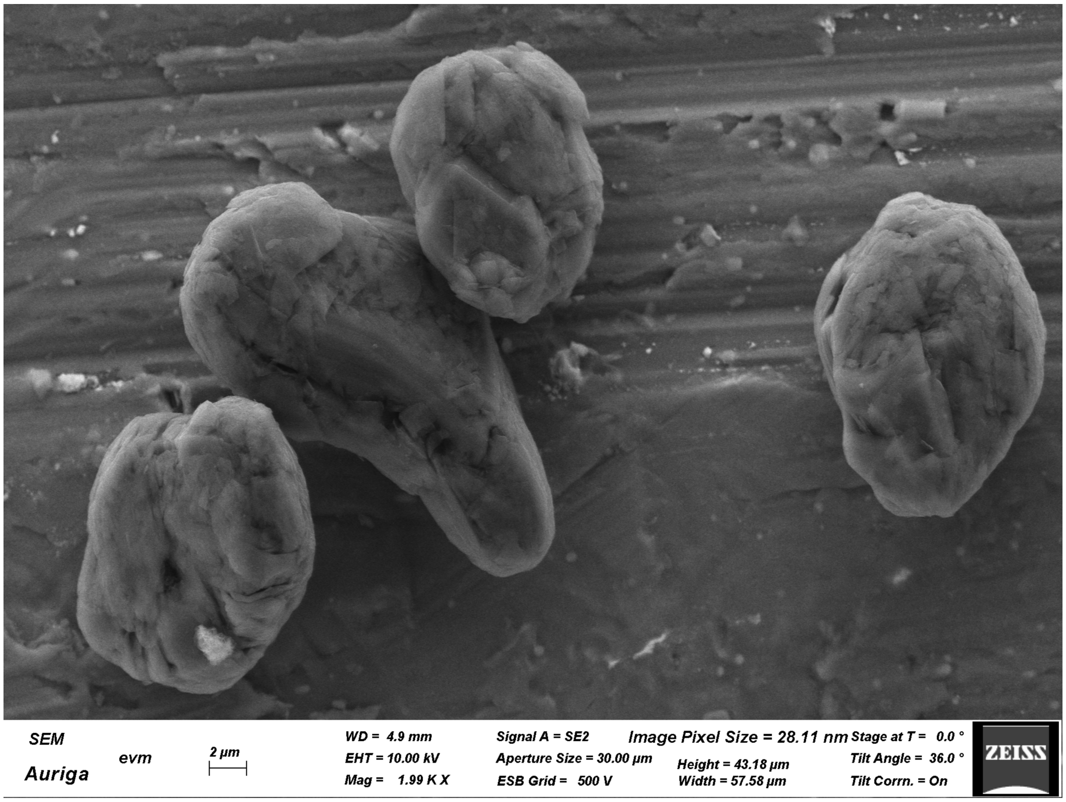


**Supplementary figure 1.** SEM picture of Actilion graphite.

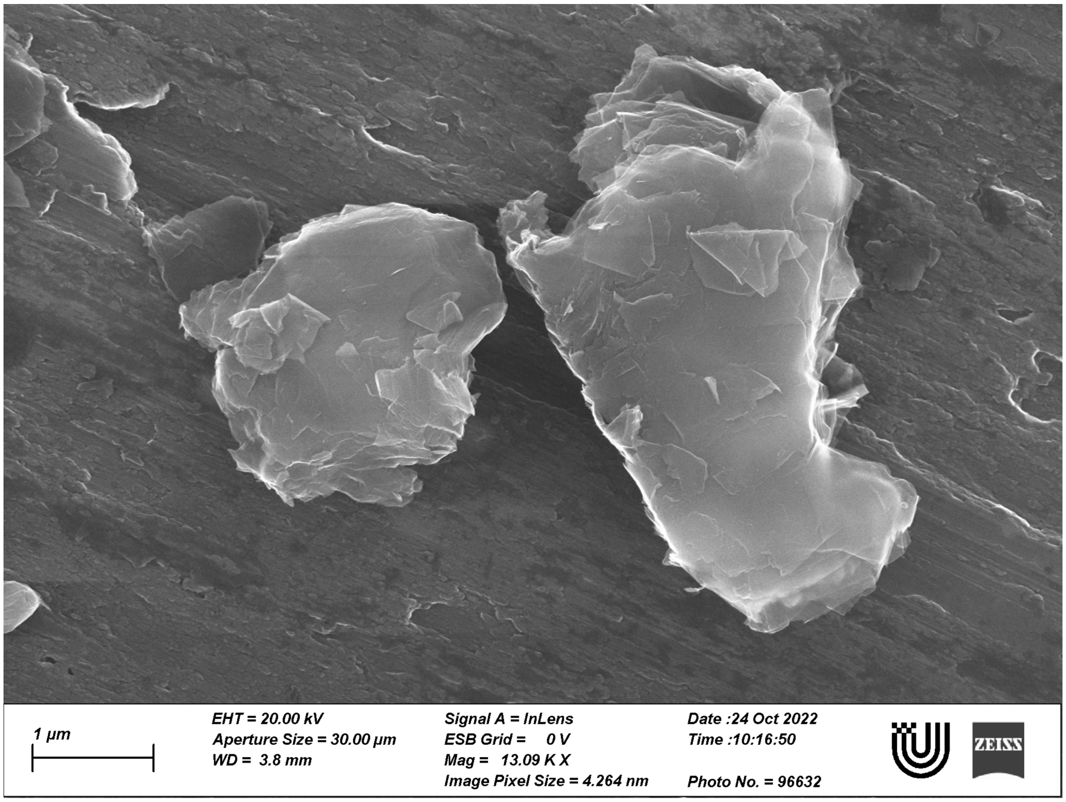


**Supplementary figure 2.** SEM picture of KS6 graphite.

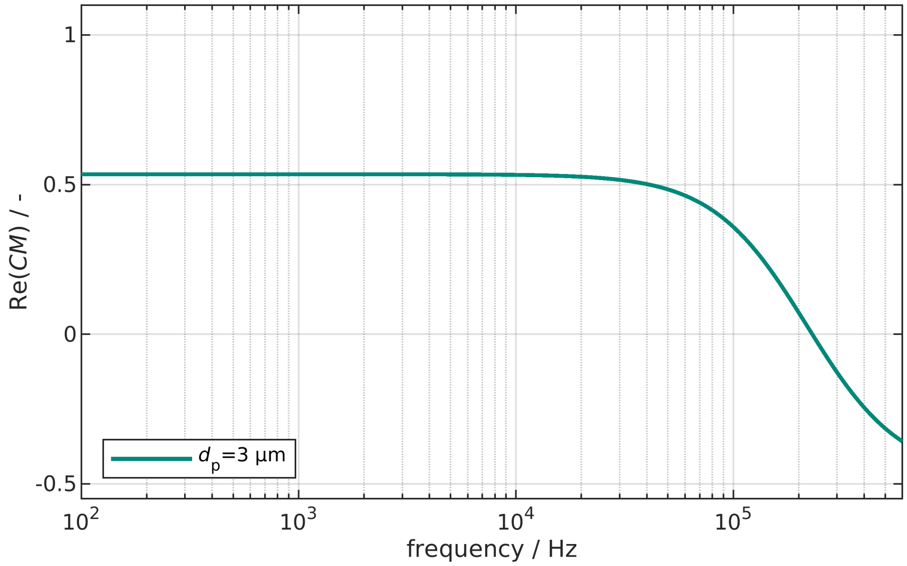


**Supplementary figure 3.** Real part of the Clausius-Mossotti factor for PS 3 µm over frequency. Surface conductance is assumed to be 1 nS and the relative permittivity 2.55. The crossover frequency ($\mathrm{Re}(CM) = 0$) is around 230 kHz. For the water a conductivity of 3 µS/cm and a relative permittivity of 78 was assumed.

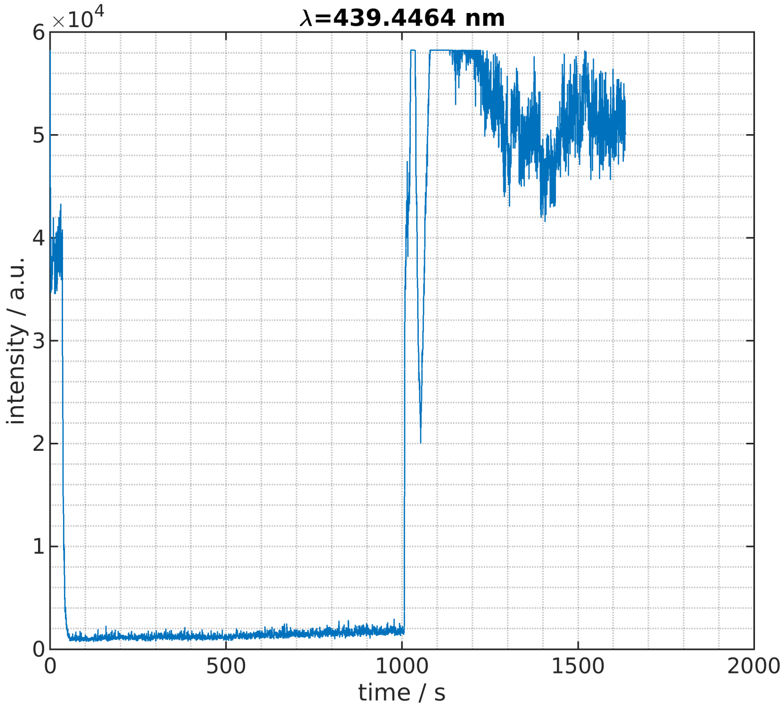


**Supplementary figure 4.** Example of the sensor signal at around 439 nm during an experiment with 800 mg/L Actilion graphite present in the solution. During remobilization ($t > 1000$ s) of the particles the maximum value of the sensor is reached and can not be measured correctly at this wavelength.

## 2 Further information on the unmixing

In Figure 5 the reference spectrum for the polystyrene (green line) and the KS6 graphite particles (blue line) are displayed. By linear combination of these two and using a global optimization the difference between this linear combination (dashed line) and the original data can be minimized. Without this unmixing step, only one signal per outlet is observable as it is displayed in Figure 6.

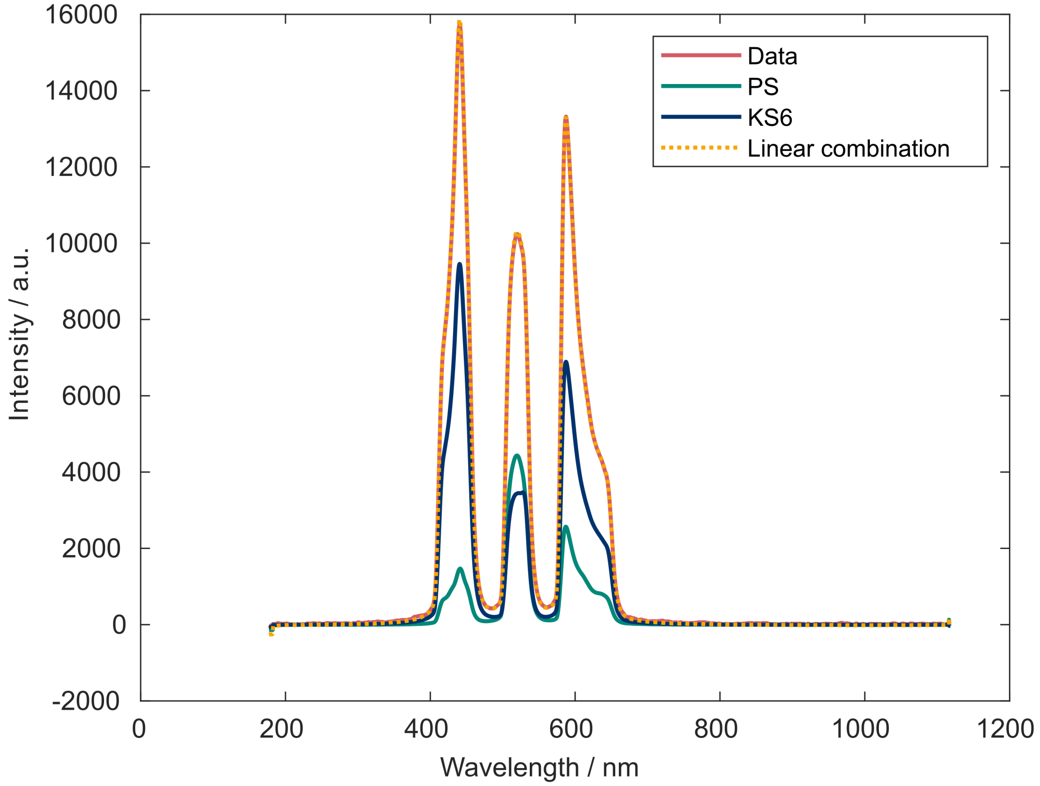


**Supplementary figure 5.** Exemplary results of the linear combination of the reference spectra of KS6 (blue line) and PS (green line) to fit the linear combination (dashed line) to the data.

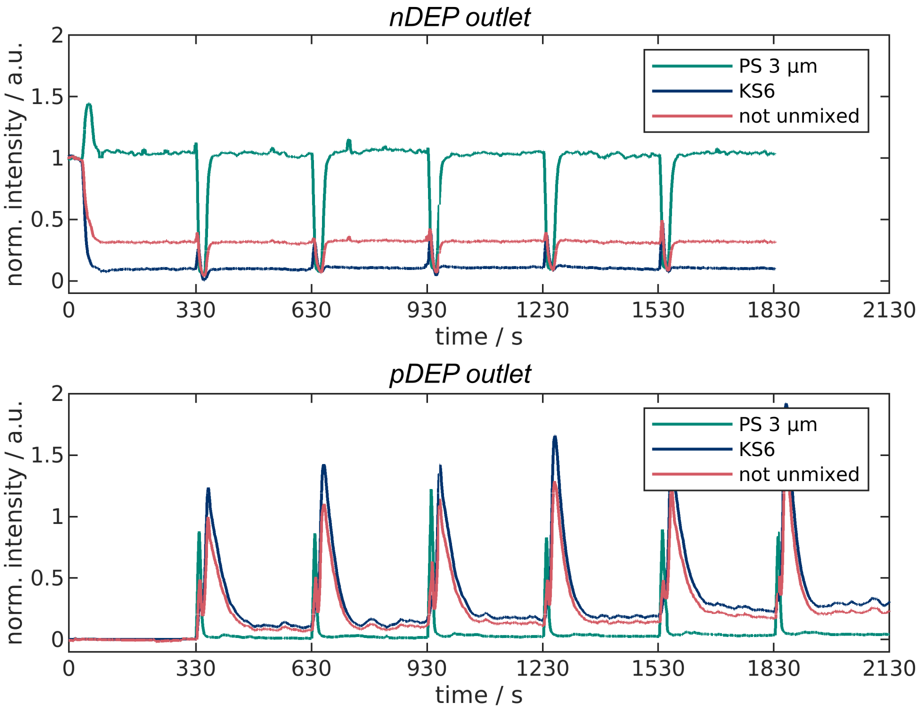


**Supplementary figure 6.** Normalized intensity over time at the *nDEP outlet* (top) and the *pDEP outlet* (bottom) for experiments with mixtures of PS or KS6 graphite present in the channels. Additionally, the data from the main document Figure 7 is displayed for comparison.
